# Effects of different feeding regimens with protease supplementation on growth, amino acid digestibility, economic efficiency, blood biochemical parameters, and intestinal histology in broiler chickens

**DOI:** 10.1186/s12917-021-02946-2

**Published:** 2021-08-25

**Authors:** Shimaa A. Amer, Rasha R. Beheiry, Doaa M. Abdel Fattah, Elshimaa M. Roushdy, Fardos A. M. Hassan, Tamer Ahmed Ismail, Noha M. A. Zaitoun, Azza M. A. Abo-Elmaaty, Abdallah E. Metwally

**Affiliations:** 1 Department of Nutrition and Clinical Nutrition, Faculty of Veterinary Medicine, Zagazig University, Zagazig, 44511 Egypt; 2 Department of Histology and Cytology, Faculty of Veterinary Medicine, Zagazig University, Zagazig, 44511 Egypt; 3 Department of Biochemistry, Faculty of Veterinary Medicine, Zagazig University, Zagazig, 44511 Egypt; 4 Animal Wealth Development Department, Faculty of Veterinary Medicine, Zagazig University, Zagazig, 44511 Egypt; 5 Department of Clinical Laboratory Sciences, Turabah University College, Taif University, P.O. Box 11099, Taif, 21944 Saudi Arabia; 6 Department of Economics, Faculty of Commerce, Zagazig University, Zagazig, 44511 Egypt; 7 Department of Pharmacology, Faculty of Veterinary Medicine, Zagazig University, Zagazig, 44511 Egypt

**Keywords:** Broiler chicken, Protease, Dried distillers’ grain with solubles, Sunflower meal, Growth performance, Ileal digestibility, Gut health

## Abstract

**Background:**

This study was conducted to estimate the impacts of using varied feeding regimens with or without protease supplementation on the growth performance, apparent amino acid ileal digestibility (AID%), economic efficiency, intestinal histology, and blood biochemical parameters of broiler chickens. Three hundred one-day-old chicks (Ross 308 broiler) were randomly allotted to a 3 × 2 factorial design. The experimental design consisted of three feeding regimens; FR1: a recommended protein SBM diet, FR2: a low-protein SBM diet, and FR3: a low-protein diet with the inclusion of 5% DDGS and 5% SFM, with or without protease supplementation (250 mg/kg).

**Results:**

Increased feed intake and feed conversion ratio were observed in the FR3 treatment during the starter stage and decreased body weight and body weight gain during the grower stage. However, there was no significant effect of the different feeding regimens, protease supplementation, or interaction on the overall performance. The economic value of diets also remained unaffected by the different feeding regimens, protease supplementation, or interaction. Protease supplementation resulted in lowering the AID% of tryptophan and leucine. Reduced AID% of methionine was evident in the FR2 + VE and FR3 − VE treatments. Histological findings substantiated the FR3 treatment mediated a decrease in the duodenal and jejunal villous height (VH), jejunal villous width (VW), and ileal VW, whereas, increase in the ileal crypt depth (CD). The FR2 + VE treatment reduced the VH:CD ratio in the duodenum. The duodenal CD and the jejunal goblet cell count were reduced as a consequence of protease supplementation. The FR3 + VE treatment documented a rise in duodenal CD, while an increase in the jejunal goblet cell count was observed in the FR3 − VE treatment. The FR3 treatment enhanced the IgM serum levels compared to the FR1 and FR2 treatments. IgM serum levels were also elevated following protease supplementation. FR3 + VE treatment increased IgM serum levels. The highest serum ALP was found in the FR3 treatment, whereas the lowest level was obtained in the FR2 treatment.

**Conclusion:**

Low-protein SBM-based diets could be used without affecting the birds’ growth. Altered morphometric measures of the intestine and increased IgM and ALP levels indicated the low-protein SBM/DDGS-SFM diet-induced damage of the intestinal histoarchitecture and immune system of birds. These different diets and protease supplementation failed to affect economic efficiency positively.

## Background

Dietary protein and amino acid balance exhibit a substantial role in gut health and performance [1]. Potential profits of low-protein diets include reducing the cost of feeding, nitrogen excretion, and environmental impact [2]. The higher utilization of nutrients by farm animals incurs a decrease in nutrient input and loss associated with the animal product and is thus beneficial. Hence, the impact of animal husbandry on the environment is reduced. Crude protein and phosphorus are environmentally related nutrients in poultry feed [3]. Cecal bacteria ferment around 10–43% of the undigested proteins [4]. Therefore, the birds’ gut health can be alleviated by reducing dietary crude protein levels and their undigested contents in the ileum or cecum. The effects of low-protein diets fortified with amino acids on broiler chickens’ growth and carcass features, estimated in several trials, reveal conflicting outcomes, leading to an ambiguous conclusion regarding the consequence of these diets on broilers’ applied production. Some studies reported no significant effect of low-protein diets on growth performance [5–7]. However, broilers fed low-protein diets (more than 3%) documented a lower growth rate and inferior carcass composition even after satisfying all nutrient requirements [8–10]. Zulkifli et al. [11] claimed reduced growth performance of broilers fed on low-protein diets under heat stress. However, low-protein diets were advantageous in enhancing survivability.

An increase in average daily gain, average daily feed intake, protein intake, energy intake, and eviscerated carcass weight was achieved by incorporating 110% Threonine in 97.5% CP of Ross recommendations. Compared to the 100% CP diets, low dietary protein encouraged abdominal fat deposition, increased serum level of uric acid, total cholesterol, and alanine aminotransferase, and decreased triglycerides [12]. Attia et al. [2] demonstrated that supplementing low-protein with amino acids improved protein utilization in finishing broilers in comparison with the high-protein diet. Moreover, no negative effects on carcass yield and breast muscle composition were witnessed by feeding a low protein diet (15% CP) and supplemented with lysine and methionine; however, there was a decrease in the nitrogen excretion by 21%.

Feed enzyme supplementation can aggravate nutrient utilization by broilers outside of a possible base for the digestive system [13, 14]. The utilization of dietary proteins and amino acids may be enhanced by protease supplementation [15, 16]. The nutritionists, therefore, preserve the growth and enhance poultry production sustainability by adding protease to low-protein diets [17]. Improved AA digestibility, feed conversion, and broiler chickens’ intestinal integrity were achieved by protease supplementation [18]. Another study confirmed that supplementing proteases may alter available substrates for bacterial growth in the gut [19]. The effects of protease supplementation are also controversial. Reports suggested an increase [18, 20, 21], decrease [22, 23], or no effect [24–26] on the AA digestibility by protease supplementation in broiler and turkey. Protease effect on the amino acid digestibility depends on its product [27] and supplementation level [20]. The feed ingredient composition directly influences the substrate and modulates other factors affecting the enzymes of the digestive system.

A massive increase in feed cost over the past decade encouraged the hunt for cost-effective feed ingredients [28]. Soybean meal has been extensively replaced by sunflower meal (SFM) as a substitute in poultry feeds [29]. Furthermore, new developments in de-hulling technology facilitated the production of a high-protein SFM, with higher crude protein (46%) and fewer crude fiber (CF, 8 to 14%) than partially de-hulled SFM (34 to 40% CP and 15 to 19% CF) and the standard (23 to 30% CP and 22 to 28% CF) [30–33]. Distiller’s dried grain with solubles (DDGS), an ethanol industry by-product, is obtained from cereal grain starch fermentation in ethanol plants. Corn, an excellent source of fermentable starch, is the primary grain used in ethanol fuel production. Corn DDGS is a rich source of amino acids, energy, minerals, water-soluble vitamins, linoleic acid, and xanthophylls for poultry feeds [34, 35]. Recent biofuel production trends have supplied nutritionists a chance to use DDGS as substitutes to protein supplements in poultry diets.

Therefore, the present study was designed to determine the effects of dietary supplementation of protease (250 mg/kg) to different feeding regimens (FR1: a recommended protein SBM diet, FR2: a low-protein SBM diet, and FR3: a low-protein diet with the inclusion of 5% DDGS and 5% SFM) on the growth performance parameters, amino acid ileal digestibility, economic efficiency, intestinal histomorphology, and blood biochemical parameters of broiler chickens.

## Results

### Growth performance

No significant interaction was observed between the different feeding regimens and protease on birds’ growth all over the experimental period (*P* > 0.05). Regardless of the protease effect, increased feed intake and FCR (*P* = 0.00) were documented by feeding birds on the FR3 treatment (low-protein SBM/DDGS-SFM diet) than FR1 and FR2 treatments during the starter period. Birds fed on the FR3 treatment revealed decreased BW and BWG compared to birds fed the FR1 treatment during the grower stage (*P* < 0.05). Different feeding regimes manifested no effect on the growth during the finisher period. The parameters all over the experimental period (*P* > 0.05) (Table 1) substantiated that, regardless of the diet effect, protease supplementation had no improving effect on the growth. No mortalities were recorded all over the experimental period among the different treatments.

**Table 1:** The effect of protease supplementation to different feeding regimens on the growth performance parameters of broiler chickens

Item	Feeding regimens			Protease level		Feeding regimens × Protease level						*P*-value			SEM
	FR1	FR2	FR3	− VE	+ VE	FR1 − VE	FR1 + VE	FR2 − VE	FR2 + VE	FR3 − VE	FR3 + VE	FR	Protease	Interaction	
Initial wt (g)	74.72	72.59	75.41	75.47	73.01	74.62	74.83	75.95	69.22	75.83	75	0.32	0.13	0.16	0.82
Starter period															
BW (g)	206.47	209.15	208.5	210.21	205.87	206.12	206.83	212.58	205.72	211.94	205.05	0.87	0.34	0.72	2.03
BWG (g)	131.7	136.56	133.08	134.74	132.85	131.5	132	136.62	136.5	136.11	130.05	0.74	0.72	0.86	2.35
FI (g)	182.25^b^	186.36^b^	224.38^a^	199.15	196.18	182.79	181.72	188.95	183.77	225.72	223.05	0.00	0.49	0.91	4.59
FCR	1.38^b^	1.37^b^	1.68^a^	1.47	1.48	1.39	1.38	1.38	1.35	1.66	1.71	0.00	0.87	0.66	0.03
Grower period															
BW (g)	897.90^a^	875.59^ab^	844.61^b^	886.62	858.77	897.91	897.88	892.29	858.88	869.66	819.55	0.02	0.05	0.33	8.51
BWG (g)	691.42^a^	666.43^ab^	636.11^b^	676.40	652.90	691.79	691.05	679.70	653.16	657.72	614.5	0.01	0.07	0.37	7.92
FI (g)	916.69	940.21	979.83	939.64	951.51	932.16	901.22	921.20	959.22	965.55	994.11	0.31	0.71	0.63	15.29
FCR	1.32	1.41	1.54	1.39	1.46	1.35	1.30	1.35	1.47	1.46	1.61	0.06	0.27	0.46	0.03
Finisher period															
BW (g)	2046.87	2009.38	2015.36	2054.17	1993.57	2062.75	2031	2054.04	1964.72	2045.72	1985	0.81	0.26	0.90	23.78
BWG (g)	1148.97	1133.79	1170.75	1167.54	1134.79	1164.83	1133.11	1161.75	1105.83	1176.05	1165.44	0.78	0.45	0.91	19.01
FI (g)	2077.17	2063.45	2144.33	2134.66	2055.31	2090.29	2064.05	2172.41	1954.5	2141.27	2147.38	0.51	0.19	0.27	29.83
FCR	1.015	1.026	1.06	1.03	1.03	1.01	1.017	1.05	0.99	1.045	1.081	0.17	0.69	0.16	0.01
Overall performance															
BW (g)	2046.87	2009.38	2015.36	2054.17	1993.57	2062.75	2031	2054.04	1964.72	2045.72	1985	0.82	0.28	0.92	23.78
BWG (g)	1972.14	1936.79	1939.94	1978.69	1920.55	1988.12	1956.16	1978.08	1895.5	1969.88	1910	0.08	0.27	0.39	23.72
FI (g)	3176.12	3190.04	3348.55	3273.46	3203.01	3205.25	3147	3282.58	3097.5	3332.55	3364.55	0.08	0.69	0.60	78.90
FCR	1.61	1.65	1.72	1.65	1.67	1.61	1.61	1.66	1.63	1.6914	1.76	0.25	0.72	0.65	0.04
PER	2.99	3.08	2.93	3.01	2.99	2.99	2.99	3.06	3.10	2.99	2.87	0.72	0.93	0.68	0.03
RGR	185.85	186.03	185.56	185.79	185.83	185.97	185.72	185.72	186.34	185.69	185.43	0.32	0.13	0.16	0.20

FR1: recommended protein corn-SBM diet; FR2: low-protein SBM diet; FR3: low-protein SBM/DDGS-SFM diet; FR1 − VE: recommended protein SBM diet without protease supplementation; FR1 + VE: recommended protein SBM diet + protease supplementation; FR2 − VE: low-protein SBM diet without protease supplementation; FR2 + VE: low-protein SBM diet + protease supplementation; FR3 − VE: low-protein SBM/DDGS-SFM diet without protease supplementation; FR3 + VE: low-protein SBM/DDGS-SFM diet + protease supplementation; BW: body weight; BWG: body weight gain; FI: feed intake; FCR: feed conversion ratio; PER: protein efficiency ratio; RGR: relative growth rate

^a,b^Means within the same row carrying different superscripts are significantly different at (*P* < *0.05*)

### Apparent ileal digestibility coefficient (AID%) of amino acids

A significant interaction between the different feeding regimens and protease level highlighted decreased AID% of methionine in the FR2 + VE and FR3 − VE treatment groups compared to the FR1 − VE treatment group (*P* = 0.006). The AID% of tryptophan was the highest in the FR1 + VE and FR3 − VE treatment groups, while, lowest in the FR2 + VE treatment group (*P* = 0.02). The AID% of lysine, threonine, arginine, leucine, isoleucine and valine were insignificantly varied among the different treatments (*P* > 0.05). Regardless of the protease effect, a decrease in the AID% of methionine, arginine, leucine, isoleucine, and valine were noted in the FR2 and FR3 treatments compared to the FR1 treatment (*P* < 0.05). Regardless of diet effect, protease supplemented groups manifested lower AID% of tryptophan and leucine than non-supplemented groups (*P* < 0.05) (Table 2).

**Table 2:** The effect of different feeding regimens, protease supplementation, or their interaction on the blood biochemical parameters of broiler chickens

Item	Feeding regimens			Protease level		Feeding regimens × Protease level						*P*-value			SEM
	FR1	FR2	FR3	− VE	+ VE	FR1 − VE	FR1 + VE	FR2 − VE	FR2 + VE	FR3 − VE	FR3 + VE	FR	Protease	Interaction	
Methionine	98.74^a^	98.69^b^	98.69^b^	98.72	98.70	98.75^a^	98.73^ab^	98.72^ab^	98.66^c^	98.68^bc^	98.71^abc^	0.006	0.06	0.01	0.007
Lysine	99.14	98.85	98.92	99.07	98.87	99.39	98.89	98.91	98.78	98.90	98.94	0.40	0.29	0.45	0.015
Threonine	98.43	98.47	98.47	98.46	98.45	98.44	98.41	98.51	98.44	98.44	98.50	0.20	0.64	0.15	0.008
Tryptophan	98.76	98.70	98.69	98.74^a^	98.69^b^	98.73^ab^	98.78^a^	98.77^a^	98.64^b^	98.71^ab^	98.67^ab^	0.06	0.04	0.02	0.015
Arginine	98.75^a^	98.68^b^	98.66^b^	98.69	98.70	98.75	98.75	98.68	98.67	98.65	98.68	0.00	0.37	0.12	0.004
Isoleucine	86.10^a^	85.68^b^	85.41^b^	85.70	85.76	86.04	86.16	85.81	85.56	85.25	85.56	0.002	0.48	0.08	0.05
Leucine	90.89^a^	90^b^	89.94^b^	90.39^a^	90.16^b^	91.05	90.74	90.11	89.88	90	89.88	0.00	0.01	0.52	0.02
Valine	98.55^a^	98.50^b^	98.46^c^	98.50	98.51	98.55	98.55	98.50	98.51	98.45	98.46	0.00	0.53	0.86	0.006

FR1: recommended protein corn-SBM diet; FR2: low-protein SBM diet; FR3: low-protein SBM/DDGS-SFM diet; FR1 − VE: recommended protein SBM diet without protease supplementation; FR1 + VE: recommended protein SBM diet + protease supplementation; FR2 − VE: low-protein SBM diet without protease supplementation; FR2 + VE: low-protein SBM diet + protease supplementation; FR3 − VE: low-protein SBM/DDGS-SFM diet without protease supplementation; FR3 + VE: low-protein SBM/DDGS-SFM diet + protease supplementation

^a,b,c^Means within the same row carrying different superscripts are significantly different at (*P* < *0.05*)

### Economic efficiency

As summarized in Table 3, the economic efficiency indicators such as feed costs, total costs, total return, net profit, feed cost/kg gain, economic efficiency, and performance index remained unaffected by the different feeding regimens, protease supplementation, or their interaction.

**Table 3:** The effect of different feeding regimens, protease supplementation, or their interaction on the economic efficiency

Item	Feeding regimens			Protease level		Feeding regimens × Protease level						*P*-value			SEM
	FR1	FR2	FR3	− VE	+ VE	FR1 − VE	FR1 + VE	FR2 − VE	FR2 + VE	FR3 − VE	FR3 + VE	FR	Protease	Interaction	
Total return (USD)/bird	2.98	2.93	2.94	2.99	2.91	3.01	2.96	2.99	2.86	2.98	2.89	0.81	0.26	0.90	0.03
Net profit (USD)	1.23	1.20	1.18	1.24	1.17	1.24	1.22	1.23	1.17	1.23	1.13	0.85	0.37	0.89	0.03
Total costs (USD)	1.75	1.72	1.75	1.75	1.73	1.76	1.74	1.75	1.68	1.74	1.76	0.49	0.28	0.40	0.01
Feed costs (USD)	1.27	1.24	1.27	1.28	1.25	1.28	1.26	1.28	1.21	1.27	1.28	0.49	0.28	0.40	0.01
Economic efficiency	0.96	0.97	0.92	0.96	0.94	0.96	0.96	0.96	0.97	0.97	0.88	0.78	0.59	0.73	0.02
Feed cost/kg gain (USD)	0.64	0.64	0.65	0.64	0.65	0.64	0.64	0.64	0.64	0.64	0.67	0.75	0.69	0.63	0.01
Performance index%	127.29	122.35	111.15	124.38	116.14	128.11	126.46	124.10	120.59	120.92	101.37	0.15	0.21	0.48	3.26

FR1: recommended protein corn-SBM diet; FR2: low-protein SBM diet; FR3: low-protein SBM/DDGS-SFM diet; FR1 − VE: recommended protein SBM diet without protease supplementation; FR1 + VE: recommended protein SBM diet + protease supplementation; FR2 − VE: low-protein SBM diet without protease supplementation; FR2 + VE: low-protein SBM diet + protease supplementation; FR3 − VE: low-protein SBM/DDGS-SFM diet without protease supplementation, FR3 + VE: low-protein SBM/DDGS-SFM diet + protease supplementation

### Histological findings of the small intestine

**Fig. 1:**
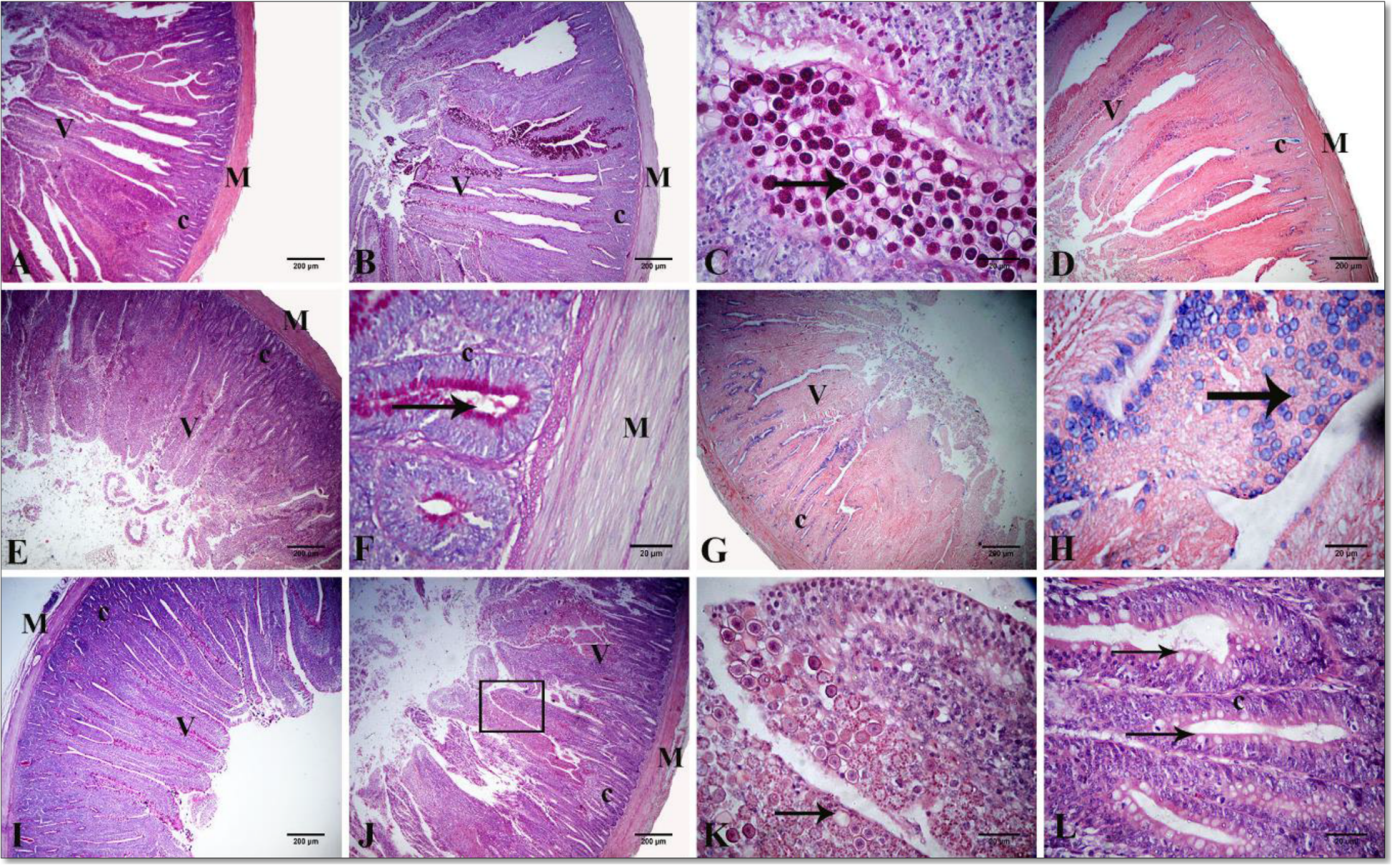
A photomicrograph of chicken intestinal tissue sections highlighting normal histological structure in cross-section of the duodenum in group I (**A**–**C**), group II (**D**), group III (**E**, **F**), group IV (**G**, **H**), group V (**I**), and group VI (**J**, **K**, **L**). Villus (V), crypt (c), goblet cells (arrows), and tunica musculosa (M). Stain: H & E in (**A**, **E**, **J**, **K**, **L**), alcian blue (**D**, **G**, **H**), and PAS in (**B**, **C**, **F**, **I**). I (FR1 − VE): recommended protein SBM diet without protease supplementation, II (FR1 + VE): recommended protein SBM diet + protease supplementation, III (FR2 − VE): low-protein SBM diet without protease supplementation, IV (FR2 + VE): low-protein SBM diet + protease supplementation, V (FR3 − VE): low-protein SBM/DDGS-SFM diet without protease supplementation, VI (FR3 + VE): low-protein SBM/DDGS-SFM diet + protease supplementation

**Fig. 2:**
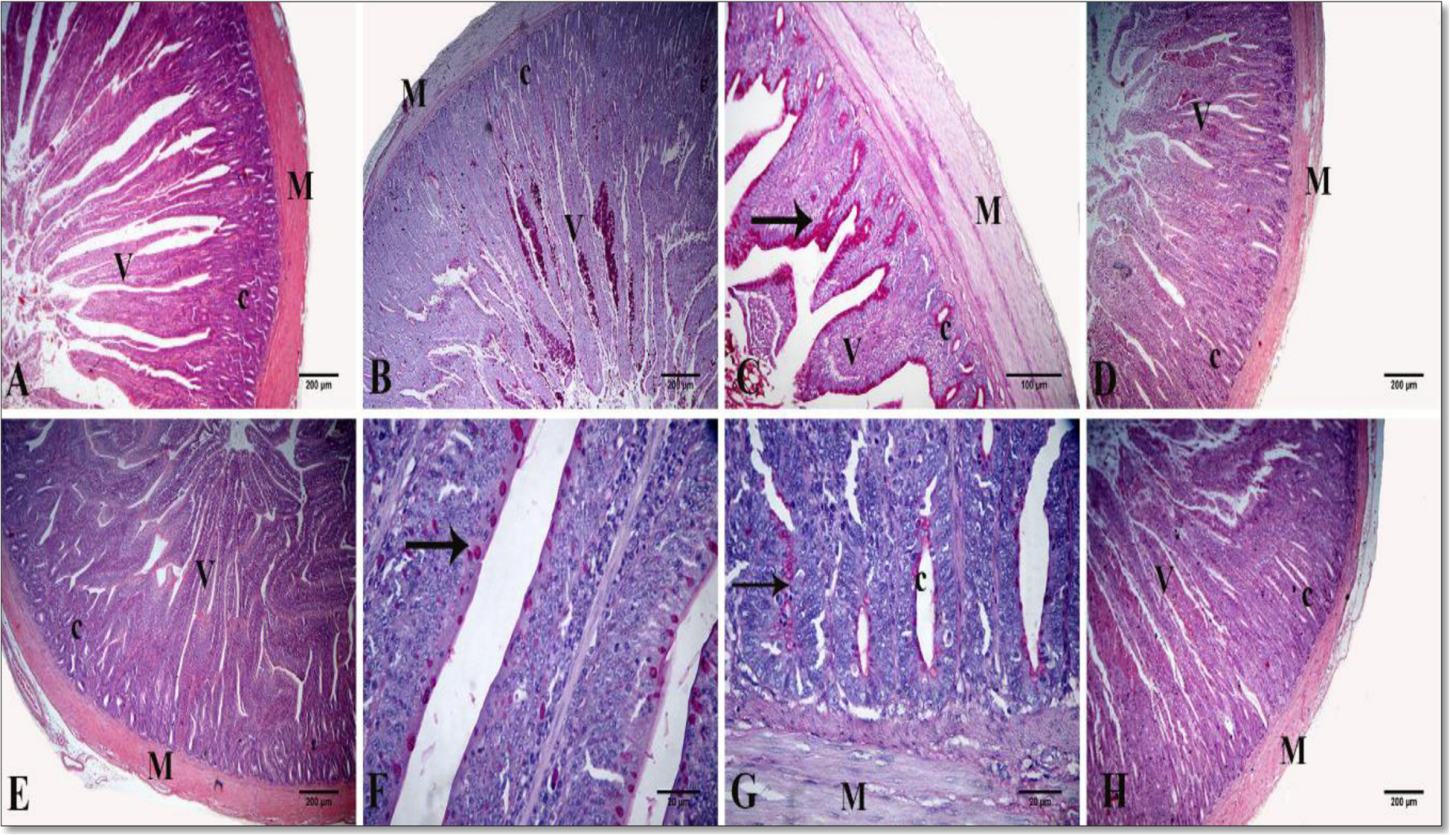
A photomicrograph of chicken intestinal tissue sections showing the normal histological structure in cross-section of jejunum in group I (**A**), group II (**B**), group III (**C**), group IV (**D**), group V (**E**–**G**), and group VI (**H**). Villus (V), crypt (c), goblet cells (arrows), and tunica musculosa (M). Stain: H & E in (**A**, **D**, **E**), and PAS in (**B**, **C**, **F**, **G**). I (FR1 − VE): recommended protein SBM diet without protease supplementation; II (FR1 + VE): recommended protein SBM diet + protease supplementation; III (FR2 − VE): low-protein SBM diet without protease supplementation; IV (FR2 + VE): low-protein SBM diet + protease supplementation; V (FR3 − VE): low-protein SBM/DDGS-SFM diet without protease supplementation; VI (FR3 + VE): low-protein SBM/DDGS-SFM diet + protease supplementation

**Fig. 3:**
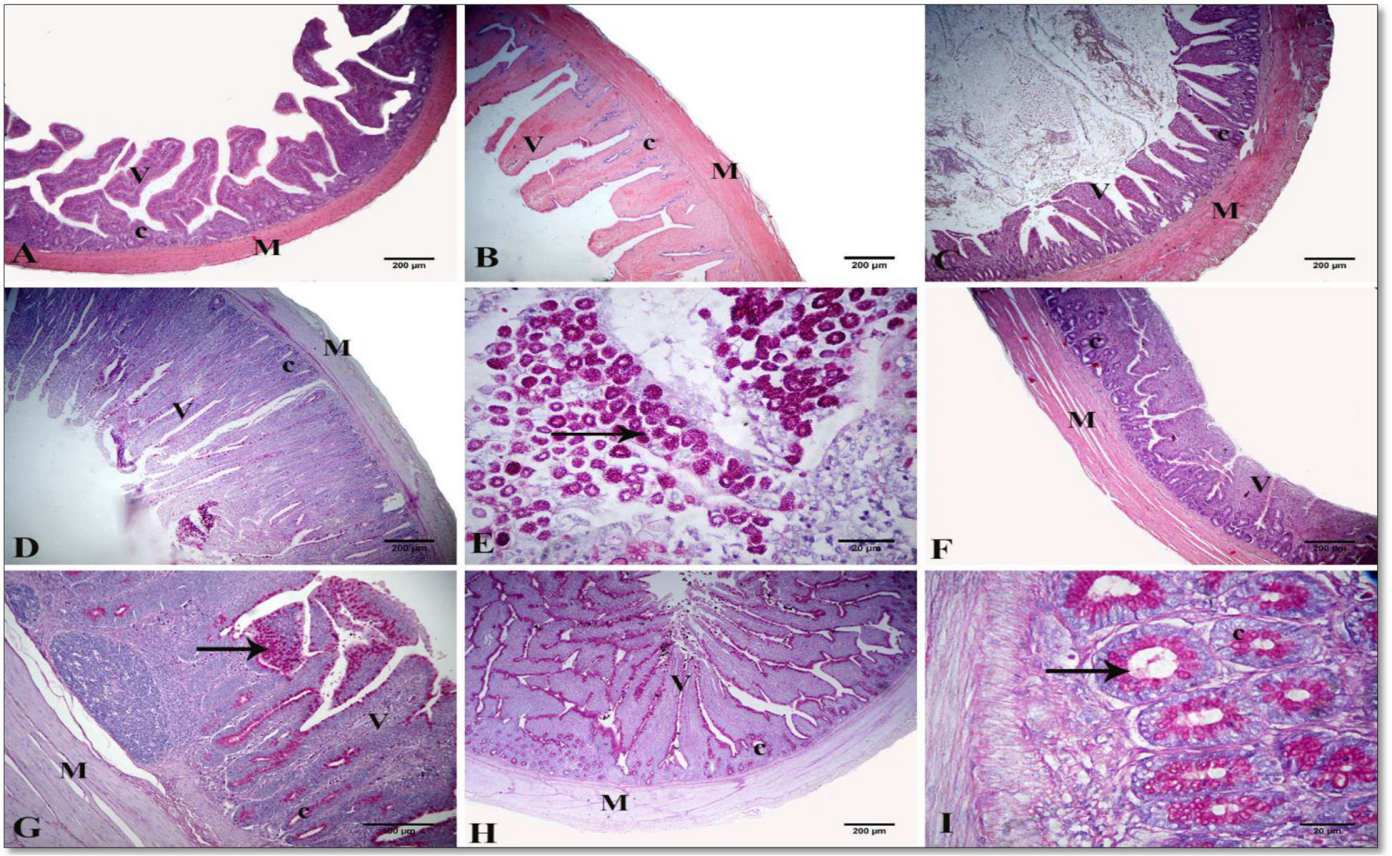
A photomicrograph of chicken intestinal tissue sections showing the normal histological structure in cross-section of ileum in group I (**A**), group II (**B**), group III (**C**), group IV (**D**, **E**), group V (**F**, **G**), and group VI (**H**, **I**), Villus (V), crypt (c), goblet cells (arrows), and tunica musculosa (M). Stain: H & E in (**A**, **C**, **F**), alcian blue (**B**), and PAS in (**G**–**I**). I (FR1 − VE): recommended protein SBM diet without protease supplementation; II (FR1 + VE): recommended protein SBM diet + protease supplementation; III (FR2 − VE): low-protein SBM diet without protease supplementation; IV (FR2 + VE): low-protein SBM diet + protease supplementation; V (FR3 − VE): low-protein SBM/DDGS-SFM diet without protease supplementation; VI (FR3 + VE): low-protein SBM/DDGS-SFM diet + protease supplementation

A standard histological structure formed from tunica mucosa, consisting of lamina epithelialis, lamina propria, and thin muscularis mucosa, was reflected in the light microscopic examination of the small intestine in the three segments for all treatments. The intestinal villi, the characteristic feature of the mucosa, differed in shape and size in each segment. The villi were nearly pointed in the duodenum, while comparatively shorter and broader in the jejunum. The villi were very short and absent in the ileum in other regions with blunt, apical, and broad basal parts. The lamina epithelialis were lined with simple columnar cells with goblet and enterochromaffin cells. The lamina propria formed from loose connective tissue contains intestinal glands or crypts, which were lined with simple columnar epithelium cells and goblet cells. The latter gave a positive reaction with PAS and alcian blue stains between the columnar lining of villi and crypts. The tunica submucosa formed from a thin layer of loose connective tissue with no Brunner’s glands. The tunica musculosa arose from thick inner circular and thin outer longitudinal of smooth muscle fibers. The tunica serosa consisted of loose connective tissue, blood vessels and lined by mesothelial cells (Figs. 1, 2 and 3).

### Morphometric measures of the small intestine

The morphometric measurements of the different parts of the small intestine of birds fed on the different treatments were illustrated in Table 4 and Figs. 1, 2 and 3. A significant interaction between the different feeding regimens and protease supplementation revealed increased duodenal crypt depth in birds fed on the FR3 + VE treatment (*P* = 0.04) and increased jejunal goblet cell count in the birds fed on the FR3 − VE treatment (*P* = 0.02). There was a reduction in the VH:CD ratio in the duodenum for the FR2 + VE treatment group (*P* = 0.03). Regardless of protease effect, birds fed on the FR3 treatment manifested decreased duodenal and jejunal villous height, jejunal VW, and ileal VW, whereas increased ileal CD (*P* < 0.05). For the FR2 treatment, a decreased VH:CD ratio in the jejunum and duodenum was witnessed (*P* = 0.02, *P* = 0.002, respectively). Regardless of diet effect, protease supplementation increased duodenal CD, decreased jejunal goblet cells, and decreased VH:CD ratio in the jejunum (*P* < 0.05).

**Table 4:** The effect of different feeding regimens, protease supplementation, or their interaction on the intestinal histomorphometric measures of broiler chickens

Item	Feeding regimens			Protease level		Feeding regimens × Protease level						*P*-value			SEM
	FR1	FR2	FR3	− VE	+ VE	FR1 − VE	FR1 + VE	FR2 − VE	FR2 + VE	FR3 − VE	FR3 + VE	FR	Protease	Interaction	
Duodenum															
VH (µm)	817.26^a^	766.05^a^	449.56^b^	711.71	643.53	821.71	812.82	821.71	710.38	491.71	407.40	0.00	0.23	0.74	47.82
VW (µm)	98.91	121.30	82.02	98.97	102.52	104.33	93.50	104.33	138.28	88.25	75.78	0.32	0.87	0.59	10.00
CD (µm)	251.83	247.74	284.26	229.95^b^	292.61^a^	241.66^b^	262.00^b^	241.66^b^	253.83^b^	206.52^b^	362.01^a^	0.39	0.02	0.04	15.73
VH/CD	3.32^a^	2.04^b^	2.25^ab^	2.82	2.25	3.49^a^	3.15^a^	2.99^ab^	1.10^b^	1.99^ab^	2.51^ab^	0.02	0.12	0.03	0.24
GCC	65.75	75.33	79.66	64.66	82.50	61.00	70.50	61.00	89.66	72.00	87.33	0.72	0.22	0.85	6.42
Jejunum															
VH (µm)	753.01^a^	666.94^ab^	360.09^b^	603.60	583.09	777.53	728.47	777.53	556.36	255.75	464.44	0.03	0.85	0.29	66.20
VW (µm)	102.03^a^	99.88^a^	59.04^b^	89.314	84.66	104.92	99.14	104.92	94.85	58.09	60.01	0.009	0.66	0.88	6.67
CD (µm)	349.40	305.51	340.41	319.81	343.73	352.22	346.59	352.22	258.79	254.99	425.82	0.90	0.78	0.44	38.33
VH/CD	2.18^ab^	1.43^b^	3.12^a^	2.66a	1.83b	2.22	2.13	2.21	0.65	3.55	2.69	0.002	0.01	0.17	0.25
GCC	58.75	65.16	77.00	78.00^a^	55.94^b^	63.00^b^	54.50^b^	63.00^b^	67.33^b^	108.00^a^	46.00^b^	0.30	0.03	0.02	6.26
Ileum															
VH (µm)	442.32	407.3	251.67	340.35	393.91	390.67	493.97	390.67	424.12	239.70	263.65	0.08	0.44	0.87	34.78
VW (µm)	111.82^a^	105.67^a^	59.83^b^	89.68	95.21	104.39	119.25	104.39	106.96	60.24	59.43	0.01	0.67	0.87	7.80
CD (µm)	110.23^b^	112.131^b^	205.48^a^	134.21	151.01	113.14	107.31	113.14	111.11	176.35	234.62	0.03	0.59	0.63	17.35
VH/CD	4.53	1.48	3.13	2.74	3.35	3.91	5.14	1.91	1.05	2.40	3.85	0.06	0.51	0.52	0.49
GCC	107.08	109.16	93.66	98.55	108.05	101.66	112.50	101.66	116.66	92.33	95.00	0.70	0.57	0.95	7.14

FR1: recommended protein corn-SBM diet, FR2: low-protein SBM diet, FR3: low-protein SBM/DDGS-SFM diet; FR1 − VE: recommended protein SBM diet without protease supplementation, FR1 + VE: recommended protein SBM diet + protease supplementation, FR2 − VE: low-protein SBM diet without protease supplementation, FR2 + VE: low-protein SBM diet + protease supplementation, FR3 − VE: low-protein SBM/DDGS-SFM diet without protease supplementation, FR3 + VE: low-protein SBM/DDGS-SFM diet + protease supplementation; VH: villous height; VW: villous width; CD: crypt depth; GCC: goblet cell count; VH/CD: villous height to crypt depth

^a,b^Means within the same row carrying different superscripts are significantly different at (*P* < *0.05*)

### Blood biochemical parameters

The serum levels of total protein, albumin, globulin, albumin/globulin ratio, and complement 3 were not significantly affected by the different feeding regimens, protease supplementation, or their interaction (*P* < 0.05). Increased IgM serum levels resulted from a significant interaction between the FR3 diet and protease supplementation (FR3 + VE) compared to other treatments (*P* = 0.04). Irrespective of the protease effect, marked elevation of IgM serum levels was observed in birds fed on the FR3 treatment compared to the FR1 and FR2 treatments (*P* = 0.01). The highest serum ALP was found in the FR3 treatment, and the lowest level was obtained in the FR2 treatment (*P* = 0.04). Regardless of the diet effect, protease supplementation increased IgM serum level (*P* = 0.04) (Table 5).

**Table 5:** The effect of protease supplementation in different feeding regimens on the blood biochemical parameters of broiler chickens

Item	Feeding regimens			Protease level		Feeding regimens × Protease level						*P*-value			SEM
	FR1	FR2	FR3	− VE	+ VE	FR1 − VE	FR1 + VE	FR2 − VE	FR2 + VE	FR3 − VE	FR3 + VE	FR	Protease	Interaction	
TP (g/dL)	7.98	7.517	8.40	7.47	8.46	7.72	8.24	7.40	7.63	7.29	9.51	0.59	0.17	0.48	0.33
Albumin (g/dL)	4.08	4.20	4.35	4.23	4.18	4.1	4.06	4.11	4.29	4.50	4.20	0.08	0.55	0.13	0.05
Globulin (g/dL)	3.69	3.31	4.05	3.17	4.19	3.44	3.94	3.28	3.34	2.79	5.30	0.65	0.13	0.29	0.32
A/G ratio (%)	1.25	1.28	1.39	1.49	1.12	1.38	1.123	1.29	1.28	1.82	0.96	0.87	0.11	0.32	0.11
ALP(U/L)	60.22ab	48.48b	74.23a	59.59	62.36	57.49	62.96	50.23	46.73	71.07	77.39	0.04	0.71	0.83	4.01
C3 (mg/dL)	109.16	99.98	117.63	101.17	116.68	110	108.33	86.51	113.46	107	128.27	0.27	0.08	0.35	4.60
IgM (mg/dL)	94.73^b^	74.36^b^	142.56^a^	87.80^b^	119.97^a^	94.77^b^	94.69^b^	73.04^b^	75.69^b^	95.59^b^	189.52^a^	0.01	0.04	0.04	10.78

FR1: recommended protein corn-SBM diet; FR2: low-protein SBM diet; FR3: low-protein SBM/DDGS-SFM diet; FR1 − VE: recommended protein SBM diet without protease supplementation, FR1 + VE: recommended protein SBM diet + protease supplementation, FR2 − VE: low-protein SBM diet without protease supplementation, FR2 + VE: low-protein SBM diet + protease supplementation, FR3 − VE: low-protein SBM/DDGS-SFM diet without protease supplementation, FR3 + VE: low-protein SBM/DDGS-SFM diet + protease supplementation; TP: total protein; A/G ratio: albumin/globulin ratio; ALP: alkaline phosphatase; C3: complement 3

^a,b^Means within the same row carrying different superscripts are significantly different at (*P* < *0.05*)

## Discussion

Dietary protein plays a significant role in digestive system development and growth performance. Modern poultry primarily focuses on reducing the feed cost to optimize economic benefits since feed is the main factor determining the total production cost, and crude protein is one of the fundamental cost constituents of poultry feed [36]. The current study documented that birds fed on a low-protein SBM/DDGS-SFM diet exhibited increased feed intake and FCR during the starter period and decreased BW and BWG during the grower stage. Reduced digestibility of most amino acids, coupled with the damage in the intestinal histomorphology observed in birds fed with a low-protein SBM/DDGS-SFM diet, could justify the above findings. The insignificant effect of dietary protease on the growth performance during the whole period may be attributed to the non-significant effect on the amino acid’s digestibility observed in our study. Furthermore, protease supplementation to the different feeding regimens failed to impart any significant effect on broiler growth, which may be explained by the protease supplements’ adverse effect on the endogenous enzyme secretion [25]. Another assumption for this result is the sufficiency of the amount of proteases present in the gut for protein digestion, or it might be related to the animal’s adaptation [37]. However, the overall growth performance remained unaffected by the interaction between the different feeding regimens (FR1: a recommended protein SBM diet, FR2: a low-protein SBM diet, FR3: a low-protein SBM/DDGS-SFM diet) and protease supplementation. Moreover, the present study also reported insignificant interaction between the feeding regimens and protease supplementation on the AID% except for the AID% of methionine that was decreased in the FR2 + VE and FR3 − VE treatments. The AID% of tryptophan was found to be highest in the FR1 + VE and FR2 − VE treatments, whereas lowest in the FR2 + VE treatment. Protease supplementation also showed no significant effect on AID% except for the AID% of tryptophan and leucine that was found to be reduced. These findings were in accordance with Siegert et al. [3], who detected no significant interaction between the source of protein and protease supplementation for nitrogen accretion and growth performance, and the outcome of the growth performance was comparable for SBM and SBM/SFM treatments. Furthermore, there was no prominent influence on the average daily gain and daily feed intake. They also claimed that the interaction between the protein source and enzyme supplementation had no effect on the prececal digestibility of crude protein and amino acids except for cystine. Lourenco et al. [38] reported that birds fed a low protein diet exhibited lower BWG and poorer overall FCR, but these parameters remained unaffected by protease inclusion. It can thus be assumed that the effect of protease on amino acid digestibility depends on the diet composition since the feed components provide the substrate for the working of the enzymes. Toghyani et al. [39] observed that supplementing diets containing SBM or SBM/canola meal with protease failed to affect the crude protein’s prececal digestibility. In agreement with these findings, Dalólio et al. [40] research also documented no effect on crude protein’s prececal digestibility by supplementing the full-fat soybeans diets with protease. Moreover, Mahmood et al. [41] found that there was no effect on prececal digestibility of crude protein by supplementing protease to different levels of poultry by-product meal replacing the SBM. When fed wheat or sorghum-based diets, the protease effect on AID% was influenced [42] and fed corn-SBM diets [16]. Besides, the difference in the dosage and protease products used may be responsible for the effect of diet ingredients used and the differences in the results. Diets were supplemented with 500 mg/kg [42] or 200 mg/kg [16, 39, 40] of protease. An earlier study revealed that protease supplementation by 1600 mg/kg increased the ileal digestibility of AA, while there was no effect on supplementation with 200 mg/kg [22].

In contrast, the same protease product’s potential was achieved after 200 mg/kg was complemented [20]. Low-CP diets-induced reduced broilers’ growth with constant ME:CP ratio was reported by Kamran et al. [43] though carcass traits were unaffected. Feeding a low-protein diet resulted in reduced nitrogen excretion that could, in turn, decrease the nitrogen loss to the environment [8, 44]. Law et al. [45] claimed that protease supplementation-mediated alleviation of the adverse effects of a low-protein diet on the broiler’s growth leads to enhanced body weight gain, FCR, and carcass traits. Mahmood et al. [46] established improved body weight gain, FCR, nutrient digestibility, nitrogen retention with no effect on the feed intake in birds fed SBM diet with 3% poultry by-product meal and supplemented with protease compared to those fed on a conventional diet (corn-SBM diet without protease supplementation). Improved amino acid digestibility by protease supplements may justify the amelioration of growth in their study without effect on the feed intake [47]. Hussain et al. [48] found that supplementation of high-protein DDGS-based diets with protease and/or enzyme blend (mannanase and xylanase) exerted no significant effect on the broilers’ growth and nutrient utilization. However, the high levels of corn-DDGS reduced growth performance [49–51]. Campasino et al. [52] informed that complementing the corn-DDGS based diets with NSPase (xylanase, glucanase, and galactosidase) exhibited no improving effect on the weight gain and FCR compared to the control diet. Protein digestibility was also found to remain unaffected by enzyme supplementation to 10% DDGS inclusion level, but the improvement was detected at 15% DDGS addition. Protease supplementation alone or in combination with amylase and xylanase amended nitrogen digestibility, as obtained by Olukosi et al. [53]. Saleh et al. [54] documented that by improving the protein digestibility, protease supplementation (200–300 mg/kg) could alleviate growth performance. Barekatain et al. [55] indicated that body weight gain and feed intake could be maintained by protease supplementation to a high inclusion level of sorghum DDGS (20%). Ndazigaruye et al. [56] validated increased BW and BWG and decreased FCR of broiler chickens by dietary protease during the starter period, while CP level has no effect on the BW and increased the FCR during the whole period. However, the interaction between CP and protease failed to significantly affect the growth performance. They concluded the relevance of dietary protease to young chicks, independent of CP levels.

Despite the similar performance of these different regimens to the control diet, there was no positive effect on the economic efficiency parameters, which may be explained by the fact that the reduced protein level was not so high to affect the diets’ cost.

The efficacy of dietary protein utilization in poultry partially relies on the digestive traits [57]. The small intestine, particularly the absorptive epithelium’s villi and crypts, plays a pivotal role in the last phase of the digestion and integration of nutrients [13, 58]. Intestinal growth can be evaluated by determining the CD, VH, and surface area, to estimate the area available for digestion and absorption [57, 59, 60]. Reports have been published on anatomical alterations in the intestine and changes in villi morphology in the different species depending on the diet type [61–63]. However, short reports highlight the correlation of the dietary nutrients’ effects, particularly protein, with the poultry gastrointestinal tract development. The presence of digested nutrients in the small intestinal lumen mostly contributes to the morphological alterations in the intestinal villi in broilers [64]. The present study confirmed the low-protein SBM/DDGS-SFM diet-induced alteration of the intestine’s morphometric measures (decreased duodenal and jejunal villous height, jejunal VW, and ileal VW, and increased ileal CD) that substantiate the reduced growth performance of birds fed on this diet during the starter and grower period and the reduced digestibility of most amino acids. The deleterious effect of low-protein diets on the intestinal morphometric measurements may be vindicated by lower concentrations of nonessential amino acids such as proline, glutamine, and glycine, effectively maintaining the epithelial layer consider a significant portion of the intestinal builders and gastrointestinal secretions [65].

Laudadio et al. [66] reported improved intestinal morphometric measures induced by a medium-protein diet (20.5% CP), resulting in enhanced broiler chicken growth performance. The absence of any significant effect on the intestinal integrity of broiler chickens was documented by Hussain et al. [48] on supplementing high-protein-based diets by protease or enzyme blend (mannanase and xylanase). The study of Buwjoom et al. [67] assessed the effect of the use of long-standing feeding of low-protein diets in broiler chickens on the intestinal villi’s histological features epithelial cells. They observed histological changes represented by long villi, large cell areas, and numerous mitotic cells in nutritional deficiency conditions, not only in hyper-nutrition conditions, which proposes that hypotrophied histological changes could indicate that the diet is nutritionally unbalanced. Barekatain et al. [68] also revealed no significant effect of dietary protein and AA levels on the VH, VW, crypt depth, and VH:CD ratio in the jejunum and ileum, although a small surface area of the jejunal villi was observed on the birds fed with a low-protein diet than those on a standard-protein diet. In accordance with these findings, no significant interaction between crude protein level and protease on the VH, VW, CD, VH:CD ratio, and intestinal absorptive surface area in the duodenum, jejunum, and ileum were highlighted by Law et al. [45]. In contrast, protease supplementation depicted an increase in the intestinal absorptive surface area. Moghaddam et al. [69] recorded reduced VH and enhanced CD in the duodenum and jejunum with increasing SFM levels.

The serum levels of TP, ALB, GL, ALB/GL ratio, and complement 3 were found to remain unaffected by the different feeding regimens, protease supplementation, or their interaction as reflected in the present study. Nonetheless, IgM serum level manifested a significant interaction in the FR3 + VE treatment group. The FR3 treatment group witnessed the highest serum ALP, while the minimal level was found in the FR2 treatment. Altered intestinal morphometric measures associated with increased serum ALP and IgM levels in birds fed the FR3 may indicate intestinal inflammation. Law et al. [45] recorded a significant interaction between crude protein level and protease on the serum levels of albumin but not for serum total protein. However, decreased serum ALB and TP were reported in broilers fed a low-protein diet. They also reported that protease supplementation failed to affect serum. TP. Saleh et al. [54] reported that protease supplementation (200–300 mg/kg) held no significant effect on serum TP. Furthermore, Perez [70] also observed that dietary inclusion of DDGS or cellulose accelerated recovery of young pigs challenged with pathogenic *Escherichia coli*. Nevertheless, this protective effect of DDGS was not detected in broilers challenged with *E. acervuline* [71]. Dietary inclusion of DDGS was found to lessen the intensity of intestinal lesions caused by *Lawsonia intracellularis* infection in young pigs, as noted by Whitney et al. [72]. Weber et al. [73] established that the expression of proinflammatory and anti-inflammatory cytokines in the intestine was up-regulated in weanling pigs fed a diet with a 7.5% DDGS inclusion level.

## Conclusion

Low-protein SBM-based diets (1% CP less than recommended by the Breeder’s Guide) could be employed without negatively impacting the birds’ growth. Owing to decreased amino acid digestibility, low-protein SMB/DDGS-SFM based diets reduced the growth performance during the starter and grower periods without influencing the overall growth performance. Altered morphometric measures of the intestine and increased IgM and ALP levels substantiated that low-protein SBM/DDGS-SFM diet may impair the intestinal histoarchitecture and immune system of birds. Protease supplementation demonstrated no constructive effect on the growth performance parameters or amino acid digestibility. There was no positive effect of these different diets and protease supplementation on economic efficiency.

## Methods

### Birds, experimental design, and diets

Three hundred one-day-old chicks (Ross 308 broiler) were obtained from a commercial chick producer (Dakahlia Poultry, Mansoura, Egypt). Before the experiments, the chicks were acclimatized to a 3-day adaptation period to reach an average body weight of 74.35 g ± 0.82 (mean ± SE). The Ethical approval of the experimental protocol was obtained from the Institutional Animal Care and Use Committee of Zagazig University, Egypt (ZUIACUC–2020). All animal experiments were performed based on the recommendations described in “The Guide for the Care and Use of Laboratory Animals in scientific investigations”. All the animal experiments also followed the ARRIVE guidelines. The trial continued for 35 days, with continuous lighting and adequate ventilation. Freshwater and feed were provided for ad libitum consumption throughout the investigation period. The chicks were reared in the same administrative, health, and environmental conditions throughout the experimental period. The routine health and vaccination practices were implemented strictly according to the recommendations. The chicks were examined daily for any health problems. After the study ended, all remaining chickens were freed.

### Experimental design and diets

Birds were randomly allotted to a 3 × 2 factorial design (5 replicates/treatment, ten chicks/replicate). The experimental design consisted of three feeding regimens; FR1: a recommended protein SBM diet, FR2: a low-protein corn-SBM diet (1% lower than recommended), and FR3: low-protein diet with the inclusion of DDGS and SFM (1% lower than recommended) with or without protease supplementation (250 mg/kg) (Protease, Cibenza^®^ EP150, Novus Europe S.A./N.V. Woluwe Atrium, Neerveld 101–103, 1200 Brussels, Belgium). The safety of Cibenza^®^ EP150 for broiler chicken has been verified [74]. Table 6 details the formulation and chemical composition of the basal diet. Following the Ross 308 broiler nutrition specifications’ standard procedures, the proximate chemical analysis of the used feedstuffs and the experimental diets was conducted [75].

**Table 6:** The proximate chemical composition of the experimental diets (%)

Ingredients	Unit	Starter stage (4–10 day)			Grower stage (11–23 day)			Finisher stage (24–35 day)		
		T1–T2	T3–T4	T5–T6	T1–T2	T3–T4	T5–T6	T1–T2	T3–T4	T5–T6
Corn 7.25% CP	%	54	56.5	52.2	58	59.60	55.30	62.52	64	59.50
Soybean meal 47% CP	%	39	37.9	31.6	32	33.80	27.50	26.00	27	21
Corn gluten meal 60% CP	%	1.2	–	–	3.20	–	–	4.50	2.24	2
Corn DDGS 26.5% CP	%	–	–	5	–	–	5	–	–	5
Sunflower meal 36% CP	%	–	–	5	–	–	5	–	–	5
Oil (Soya)	%	2	1.55	2.20	3	2.90	3.5	3.50	3.30	4
Dicalcium phosphate 18%	%	2	2	1.80	1.70	1.70	1.55	1.45	1.45	1.33
Calcium carbonate	%	0.5	0.40	0.40	0.50	0.45	0.50	0.50	0.45	0.50
Dl methionine 99%	%	0.39	0.35	0.33	0.28	0.31	0.29	0.27	0.30	0.28
Sodium bicarbonate	%	0.32	0.37	0.31	0.33	0.32	0.31	0.32	0.30	0.30
Broiler Premix^a^	%	0.3	0.30	0.40	0.30	0.30	0.30	0.30	0.30	0.30
l-LYSINE Hcl 98%	%	0.25	0.29	0.32	0.30	0.25	0.37	0.28	0.27	0.38
Salt	%	0.11	0.08	0.07	0.11	0.12	0.08	0.13	0.13	0.10
l-Threonine 98.5%	%	0.10	0.09	0.13	0.10	0.07	0.09	0.06	0.08	0.10
Choline	%	0.06	0.07	0.10	0.07	0.07	0.10	0.07	0.07	0.10
Antimycotoxin	%	0.1	0.1	0.10	0.10	0.10	0.10	0.10	0.10	0.10
Phytase enzyme	%	0.005	0.005	0.005	0.005	0.005	0.005	0.005	0.005	0.005
Chemical analysis										
Moisture	%	11.35	11.44	11.19	11.23	11.32	11.07	11.18	11.25	11.00
Crude protein	%	23.2	22.20	22.22	21.55	20.53	20.56	20.15	19.10	19.10
Crude fat	%	4.91	4.49	5.53	6.04	5.87	6.85	6.67	6.43	7.50
Lysine	g/kg	14.48	13.71	13.77	13.20	13.05	13.05	11.55	11.57	11.55
Methionine	g/kg	7.34	6.76	6.73	6.18	6.14	6.12	5.97	6.02	5.97
Methionine + cystine	g/kg	10.88	10.37	10.22	9.58	9.34	9.40	9.19	9.08	9.12
Threonine	g/kg	9.85	9.41	9.62	9.13	8.52	8.55	8.03	7.96	7.99
Tryptophan	g/kg	2.83	2.75	2.63	2.49	2.50	2.38	2.18	2.17	2.07
Arginine	g/kg	15.53	15.09	14.79	13.72	13.76	13.49	12.07	12.02	11.80
Valine	g/kg	11.10	11.04	11.08	10.27	10.14	10.23	9.45	9.07	9.01
Calcium	g/kg	8.79	8.43	8.43	7.83	7.70	7.68	7.09	6.95	7
Av. Phosphorus	g/kg	4.98	4.98	4.91	4.47	4.49	4.49	4.03	4.04	4.09
Sodium	g/kg	1.59	1.59	1.59	1.62	1.61	1.61	1.67	1.60	1.67
Potassium	g/kg	9.19	9.11	8.87	8.05	8.41	8.19	7.07	7.29	7.11
Cl	g/kg	2.28	1.98	2.25	2.32	2.30	2.36	2.34	2.33	2.43
Crude fiber	%	3.52	3.52	4.29	3.23	3.34	4.11	2.99	3.05	3.84
ME (kcal/kg)	Kcal/kg	2991.12	2969.7	2973.74	3103.42	3071.17	3070.72	3200.15	3173.76	3173.84

T1 (FR1 − VE): recommended protein SBM diet without protease supplementation; T2 (FR1 + VE): recommended protein SBM diet + protease supplementation; T3 (FR2 − VE): low-protein SBM diet without protease supplementation; T4 (FR2 + VE): low-protein SBM diet + protease supplementation; T5 (FR3 − VE): low-protein SBM/DDGS-SFM diet without protease supplementation; T6 (FR3 + VE): low-protein SBM/DDGS-SFM diet + protease supplementation

^a^Premix per kg of diet: vitamin A, 1 500 IU; vitamin D3, 200 IU; vitamin E, 10 mg; vitamin K3, 0.5 mg; thiamine, 1.8 mg; riboflavin, 3.6 mg; pantothenic acid, 10 mg; folicacid, 0.55 mg; pyridoxine, 3.5 mg; niacin, 35 mg; cobalamin, 0.01 mg; biotin, 0.15 mg; Fe, 80 mg; Cu, 8 mg; Mn, 60 mg; Zn, 40 mg; I, 0.35 mg; Se, 0.15 mg

### Growth performance

The average initial body weight was recorded on the 4th day of age, and then the body weight was recorded at 10, 23, 35 days.

The body weight gain (g/bird) = W2 − W1, where W2 is the final body weight at the intended period, and W1 is the initial body weight in the same period.

$${\text{Feed intake}}\left( {{\text{g}}/{\text{bird}}} \right) = {\text{feed offered weight}} - {\text{residues left}}/{\text{birds\ No}}.$$

The feed conversion ratio was estimated weekly: FCR = the amount of feed consumed (g)/Bodyweight gain (g).

The relative growth rate (RGR) was calculated using the equation described by [76].

$${\text{RGR}} = {\text{W}}2{-}{\text{W}}1/\raise.5ex\hbox{$\scriptstyle 1$}\kern-.1em/ \kern-.15em\lower.25ex\hbox{$\scriptstyle 2$} {\text{ }}\left( {{\text{W}}1 + {\text{W}}2} \right) \times 100.$$

W1: the initial live weight (g), W2: the live weight at the end of the considered period (g).

Protein efficiency ratio (PER) was determined according to [77].

$${\text{PER}} = {\text{Live weight gain}}\left( {\text{g}} \right)/{\text{Protein intake}}\left( {\text{g}} \right).$$

### Amino acids ileal digestibility

The amino acids’ ileal digestibility was determined by estimating Titanium dioxide, an indigestible indicator substance, as described by Amer et al. [13]. The amino acid concentration in the diet and ileal digesta samples were evaluated according to Li et al. [78] and Siriwan et al. [79]. Tryptophan was ascertained separately, according to Ravindran and Bryden [80]. Titanium dioxide was estimated following the procedures delineated by Fenton and Fenton [81].

$${\text{AID}}\left( \% \right) = {\text{1}}00 - \left[ {\left( {{\text{Ti }}\left( {{\text{diet}}} \right) \times {\text{AA}}\left( {{\text{ileum}}} \right)} \right)/\left( {{\text{TI}}\left( {{\text{ileum}}} \right) \times {\text{AA}}\left( {{\text{diet}}} \right)} \right) \times {\text{1}}00} \right]$$

Ti (diet): titanium dioxide concentration in the diet. Ti (ileum): titanium dioxide concentration in ileal digesta. AA (ileum): the concentration of the test AA in ileal digesta. AA (diet): the concentration of the test AA in the diet.

### Economic efficiency

Collective efficiency measures, which include total return, total costs, variable costs, and net profit, were calculated according to [82, 83].

Total feed cost (USD/bird) = Total feed intake/bird × Price of 1 kg feed.

Total cost (USD/bird) was computed by considering feed cost as well as the expenses of 1-day-old chick, litter, labor, veterinary services, electricity, and other miscellaneous expenditure, that were common to all groups.

Total return (USD/bird) = Live body weight/bird × Price of kg body weight.

Net profit (USD/bird) = Total returns − Total costs.

Economic efficiency (E.EF) = Net profit/Total feed cost.

Feed cost/kg gain (USD/bird) = Total feed cost/Total weight gain.

The performance index (PI) was calculated based on a previous study [84].

Performance index % (PI) = final live body weight (kg)/feed conversion × 100.

### Sample collection and laboratory analyses

At the end of the experiment, five randomly chosen birds per treatment were euthanized using cervical dislocation, according to the American Veterinary Medical Association guidelines [85], and blood samples were collected. Samples were left to coagulate at 4 °C and centrifuged at 3500 rpm for 15 min to extract the serum, which was then stored in Eppendorf tubes at – 20 °C until being analyzed. Samples from different parts of the small intestine were obtained for histological examination.

The serum concentration of total proteins was determined colorimetrically using the biuret method [86]. The serum levels of alkaline phosphatase and IgM were determined using chicken ELISA kits of MyBioSource Co. of CAT.NO. MBS012469, MBS701683, and of ABCAM Co. of CAT. NO. AB157691, respectively. A sandwich enzyme-linked immunosorbent assay (ELISA) kit manufactured by Life Span Biosciences, Inc. of CAT.NO.LS-F9287 was employed to estimate the serum complement 3 levels by following the manufacturer’s instructions.

### Histological examination of the small intestine

The tissue samples (approximately 1 cm) were obtained from the midpoints of the three segments of the small intestine (duodenum, jejunum, and ileum), gently washed with normal saline to remove any debris, and then fixed in 10% buffered neutral formalin. The tissue samples were subjected to routine histological processing and embedded in paraffin. Finally, 5–7 micron-thick sections were cut and stained by Harris’s hematoxylin and Eosin to elucidate the general structures, Periodic acid Schiff technique for neutral and some acidic mucopolysaccharides, Alcian blue pH (2.5) for detecting acidic mucopolysaccharides. The methods of processing and staining were adopted following [87]. Image J software (http://Sb.Info.nih.gov/ij/) was applied for performing the measurements, including, Intestinal villi length (from the upper region to the junction between the villus and crypt), villi width, and crypts depth (from the base of the villi to muscularis mucosa) by examination of five different nonoverlapping fields in three separate H&E-stained sections of different birds in each group on low power field (40× magnification) while the number of goblet cells was on power field (400× magnification). The density of goblet cells was calculated as the number of goblet cells per unit of surface area (mm^2^).

### Statistical analysis

Shapiro–Wilk’s test was employed to verify the normality, and Levene’s test was exploited to ascertain the homogeneity of variance components between experimental treatments, and the assumption was achieved (*P* > 0.05). Variations were assessed by two-way (ANOVA), and factorial analysis was executed on the factors included in the model, such as feeding regimen, protease supplementation, and their interaction. The differences between the means were compared by the post-hoc Tukey’s multiple range tests at 5% probability. Variation in the data was expressed as pooled SEM. The significance level was set at *P* < 0.05.

## Data Availability

The datasets used and analyzed during the current study available from the corresponding author on reasonable request.

## Abbreviations

AID%: Amino acid ileal digestibility coefficient
BW: Body weight
BWG: Body weight gain
FI: Feed intake
FCR: Feed conversion ratio
RGR: Relative growth rate
PER: Protein efficiency ratio
Ti: Titanium dioxide
AA: Amino acid
